# Susceptibility-Guided Therapy vs. Bismuth-Containing Quadruple Therapy as the First-Line Treatment for *Helicobacter pylori* Infection: A Systematic Review and Meta-Analysis

**DOI:** 10.3389/fmed.2022.844915

**Published:** 2022-03-24

**Authors:** Yaobin Ouyang, Wenjing Zhang, Chen He, Yin Zhu, Nonghua Lu, Yi Hu

**Affiliations:** ^1^Department of Gastroenterology, The First Affiliated Hospital of Nanchang University, Nanchang, China; ^2^Medical College of Nanchang University, Nanchang, China; ^3^Department of Nephrology, The First Affiliated Hospital of Nanchang University, Nanchang, China

**Keywords:** *Helicobacter pylori*, susceptibility-guided therapy, bismuth containing quadruple therapy, efficacy, meta-analysis

## Abstract

**Background:**

The increased antibiotic resistance of *Helicobacter pylori* (*H. pylori*) has led to the decreased efficacy of *H. pylori* regimens.

**Aim:**

To evaluate the efficacy, safety, and compliance of susceptibility-guided therapy (SGT) vs. bismuth-containing quadruple therapy (BQT) as the first-line treatment for *H. pylori* infection.

**Materials and Methods:**

This meta-analysis was performed in accordance with the PRISMA 2009 guidelines. A systematic search in PubMed, Embase, and Cochrane databases was conducted using the combination of “*H. pylori* or *H. pylori* or *Hp*,” “bismuth quadruple,” and “tailored eradication OR tailored therapy OR susceptibility-guided therapy OR personalized therapy OR antibiotic susceptibility testing.”

**Results:**

Five studies with 2,110 *H. pylori-*infected patients were enrolled. The pooled eradication rates of SGT and BQT were 86 vs. 78% (*p* < 0.05) and 92 vs. 86% (*p* > 0.05) by intention-to-treat (ITT) and per-protocol (PP) analyses, respectively. SGT has a significantly superior efficacy than BQT [pooled risk ratio (RR) = 1.14, *p* < 0.05] in a subgroup of cultures with the susceptibility test. The pooled side effect rate was 20% in SGT and 22% in BQT, which showed no significant difference (*p* > 0.05). The compliances of SGT and BQT were 95 and 92%, respectively.

**Conclusion:**

Compared with BQT, SGT showed a higher efficacy and similar safety as the first-line treatment of *H. pylori* infection in areas with high antibiotic resistance. The decision-making of first-line regimens for *H. pylori* infection should depend on the availability and cost-effectiveness of susceptibility tests and bismuth in local areas.

## Introduction

*Helicobacter pylori* (*H. pylori*) is acquired in childhood and can be transmitted person-to-person *via* oral–oral, fecal–oral, or gastro–oral transmission routes (1). *H. pylori* gastritis was defined as an infectious disease in the Kyoto global consensus report (2). As a type-1 carcinogen, *H. pylori* infection is closely associated with an incidence of gastric cancer (GC) (3). Due to the public health burden and threat caused by *H. pylori* infection and the thought that successfully clearing *H. pylori* could eliminate a carcinogen and reduce a source of infection, recent consensus reports have raised the importance of *H. pylori* screening and eradication for GC prevention (4, 5). They have also demonstrated that family-based *H. pylori* infection control and management are suitable approaches to prevent intrafamilial transmission and reduce the incidence of *H. pylori*-related diseases (6).

Widespread use of antibiotics leads to increased antimicrobial resistance, and the resistance rate of common antibiotics (clarithromycin, etc.) used in *H. pylori* regimens was reported to be more than 15% (7). Moreover, clarithromycin-resistant *H. pylori* was included in a high-priority tier for investment in new drugs (8). Compared with treatment-naive adults, previously treated adults showed a higher antibiotic resistance rate (9). Therefore, elevating the first-line efficacy of the *H. pylori* regimen is important to avoid secondary antibiotic resistance. Non-bismuth-containing quadruple therapies (including concomitant, sequential, and hybrid) are all influenced by dual antibiotic resistance (10). High-dose dual therapy has the advantages of less unnecessary antibiotic use and fewer side effects and shows a similar efficacy in comparison with the current mainstream guideline-recommended therapies (11), which is an alternative treatment for *H. pylori* infection. Bismuth-containing quadruple therapy (BQT) is currently the first-line treatment for *H. pylori* infection because it achieves a high efficacy in susceptible and resistant *H. pylori* strains (4, 12, 13).

Theoretically, any therapy for infectious diseases should be based on the results of susceptibility testing. Susceptibility-guided therapy (SGT) is an effective way to achieve high efficacy, have limited side effects, and to avoid unnecessary antibiotic use. Real-time PCR is a rapid method to diagnose *H. pylori* infection and detect the point mutations of clarithromycin, which guides the antibiotic choice for *H. pylori* eradication (14). However, the availability, accuracy, and cost-effectiveness of SGT should be considered in the clinical management of *H. pylori* infection (15). The *H. pylori* consensus reports have also demonstrated inconsistent statements regarding the application of SGT in the eradication of *H. pylori* (4, 5, 13, 16). Several clinical trials (17–19) were conducted to evaluate whether SGT (based on susceptibility testing or PCR results) shows a superior or similar efficacy in comparison with BQT.

Hence, this systematic review and meta-analysis were conducted to evaluate the efficacy, side effects, and compliance of SGT as the first-line treatment for *H. pylori* in comparison with BQT (the current mainstream guideline-recommended therapy), guiding the clinical choice of *H. pylori* regimens, especially in areas with high clarithromycin resistance.

## Methods

### Search Strategy

This systematic review and meta-analysis were registered in PROSPERO (registration no.: CRD42022284258) and performed in accordance with the PRISMA 2009 guidelines (20). Relative records were searched through PubMed, Embase, and Cochrane (Cochrane Central Register of Controlled Trials) databases. The following search terms were used: “*Helicobacter pylori* or *H. pylori* or *Hp*,” “bismuth quadruple,” and “tailored eradication OR tailored therapy OR susceptibility-guided therapy OR personalized therapy OR antibiotic susceptibility testing.” Only English articles published from 1983 to December 13, 2021 were evaluated. The search strategy for each database is shown in detail in Supplementary Table S1.

### Study Selection

Two researchers (OY and ZW) independently evaluated all relevant records in two stages, and any disagreement was discussed until a consensus was reached; otherwise, the decision was made by a third researcher (HC). In the first stage, irrelevant records were excluded by evaluating their titles and abstracts. In the second stage, the full text of the remaining studies was assessed with the following criteria. Inclusion criteria: P (Patients), *H. pylori*-infected adult patients without a history of *H. pylori* eradication; I (Intervention), SGT, defined as a consensus recommended triple and/or quadruple therapy based on the antimicrobial susceptibility test to clarithromycin and/or metronidazole and/or levofloxacin and other antibiotics (4); C (Comparator), empiric BQT recommended by the current guidelines (4, 12); O (Outcome), the eradication rate, side effects and compliance of SGT and BQT; S (Study design), only randomized controlled trials (RCTs) were included. The exclusion criteria were as follows: studies in children or the elderly, basic studies, case reports, conference abstracts, reviews, meta-analyses, and studies with insufficient published data.

### Data Extraction and Quality Assessment

Two investigators (OY and ZW) independently extracted the data from the included studies, and discrepancies were fully discussed until an approval was reached; otherwise, the decision was made by a third investigator (HC). The following data were extracted from the included studies: study year of publication; first author; country; patient characteristics; tests to confirm *H. pylori* infection and eradication (including the time interval between an end of the treatment and the confirmation of successful *H. pylori* eradication test); eradication regimen, *H. pylori* eradication rate (intention-to-treat analysis, ITT; per-protocol analysis, PP), side effects, and the compliance of SGT and BQT.

The methodological quality of the enrolled studies was assessed. RCTs were assessed by the Cochrane Risk of Bias Assessment Tool for the following categories: selection bias, performance bias (4), detection bias, attrition bias, reporting bias, and other bias (21).

### Endpoint

The primary endpoint of this study was to compare the eradication rate of SGT and BQT by the ITT and PP analysis, and the secondary endpoint was to compare the side effects and the compliance of both groups.

### Statistical Analysis

This meta-analysis was performed by Stata software (version 12; Stata Corp LP), and *p* < 0.05 was regarded as a significant difference. Risk ratios (RRs) and corresponding 95% CIs were used to determine the effect of SGT and BQT. A random effect model was used to reduce the potential bias caused by heterogeneity. Heterogeneity was detected by the chi-squared test and *I*^2^ test, with the value of *p* < 0.10 of the chi-squared test or *I*^2^ > 50% defined as a significant heterogeneity. Publication bias was estimated by a funnel plot, Begg's test, and Egger's test. The value of *p* < 0.05 of Begg's test and Egger's test was deemed to indicate a significant publication bias. When heterogeneity was large, studies were removed one by one, and changes in heterogeneity were observed to find the source of heterogeneity.

## Results

### Study Selection and Characteristics

**Figure 1:**
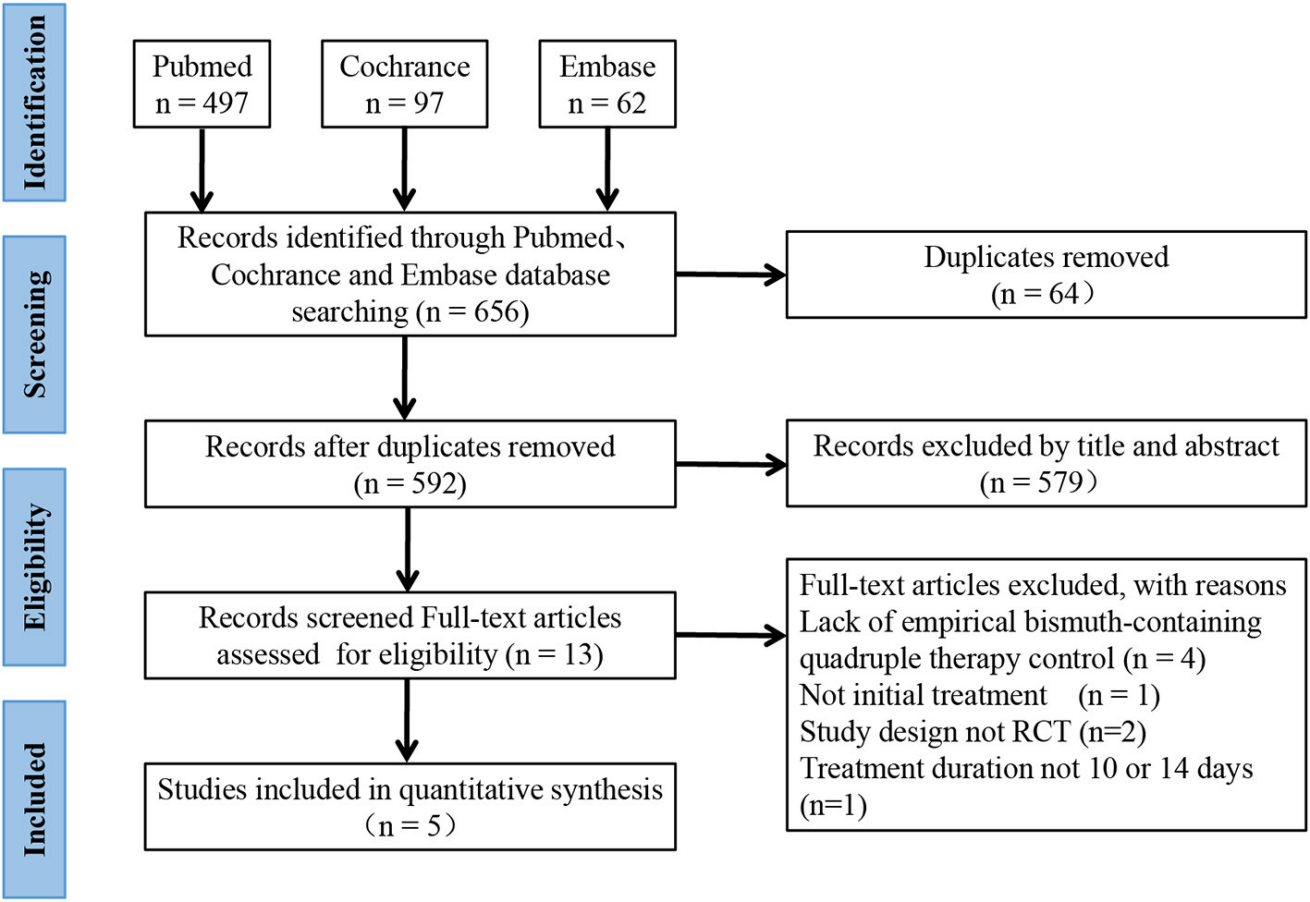
A flowchart of the details of this study.

As shown in Figure 1, 656 records were identified, and 64 were removed due to duplication. In the first stage, 579 studies were removed after the title and abstract screening. In the second stage, 13 studies were reviewed by full text, and 8 studies were removed for the lack of empirical BQT control group (*n* = 4), lack of initial treatment (*n* = 1), a non-RCT study design (*n* = 2), and a treatment duration not equal to 10 or 14 days (*n* = 1). Finally, 5 studies (17–19, 22, 23) and 2,110 *H. pylori-*infected patients were enrolled; 1,100 patients were assigned to SGT, and 1,010 were assigned to BQT.

The study characteristics are shown in Table 1. All of the studies were published between 2015 and 2021; 4 studies were conducted in China, and 1 was conducted in Korea. In these 5 studies, antimicrobial susceptibility was determined by the *E*-test in 2 studies, the agar-dilution test in 2 studies, and the dual-priming oligonucleotide-based multiplex PCR (DPO-PCR) test in 1 study. Susceptibility-based triple and/or quadruple therapy was used in the SGT group in all studies.

**Table 1:** Characteristics of the studies included in the meta-analysis.

						**Test for confirming**			**Antibiotic resistant rate**		**Eradication regime**		**Eradication rate**			
						***H. pylori***										
**Year**	**First author**	**Region**	**Study design**	**Patients characteristic**	**Patients number**	**Infection**	**Eradication (time interval)**	**Antibiotic Susceptibility Testing**	**Susceptibility-guided therapy**	**Bismuth-containing quadruple therapy**	**Susceptibility-guided therapy (SGT)**	**Bismuth-containing quadruple therapy (BQT)**	**ITT**	**PP**	**Side effects**	**Compliance**
2015	Liya Zhou	China	RCT	*H. pylori* infection	668	Both RUT and W-S staining	UBT (4–12 weeks)	E-test	Cla 46.2% (147/350), Met 65.4% (208/350), Amo 1.2% (4/350)	Cla 52.4% (166/350), Met 68.8% (218/350), Amo 3.2% (10/350)	Triple therapy according to Cla sensitivity test. and CYP2C19 genotyping results	Esomeprazole 20 mg, bismuth potassium potassium citrate 220 mg, Amo 1,000 mg, Cla 500 mg, bid for 10 days	TT: 88.7% (282/318) BQ: 77.4% (271/350)	TT: 93.3% (278/298) BQ: 87.0% (261/300)	TT: 22% (70/318) BQ: 26.6% (93/350) 26.6% (93/350)	TT: 94.3% (300/318) BQ: 90% (315/350)
2015	Fangyuan Dong	China	RCT	*H. pylori* positive patients with dyspepsia complaint	90	C	UBT (4–6 weeks)	E-test	Cla 40.0% (18/45), Met 53.3% (24/45), Lev 55.6% (25/45), Amo 4.4% (2/45), TCY 0% (0/45)	NA	Quadruple therapy based on E-test results twice daily for 14 days	Rabeprazole 10 mg, Bismuth potassium citrate 600 mg, Amo 1,000 mg and Cla 500 mg, bid for 14 days	TT: 91.1% (41/45) BQ: 73.3% (33/45)	TT: 95.3% (41/43) BQ: 78.6% (33/42)	TT: 2.2% (1/45) BQ: 4.4% (2/45)	NA
2019	Jie Pan	China	RCT	*H. pylori* positive patients and upper gastrointestinal symptoms	467	Both UBT and C	UBT (8 weeks)	Agar-dilution test	Cla 28.7% (89/310), Met 96.5% (299/310), Lev 30.6% (95/310), Amo 0% (0/310), Fur 0% (0/310)	Cla 21.02% (33/157), Met 97.45% (153/157), Lev 24.84% (39/157), Amo (33/157), Met 97.45% (153/157), Lev 24.84% (39/157), Amo 0% (0/157), Fur 0% (0/157)	Triple therapy or Bismuth-quadruple therapy based on the antibiotic susceptibility test results for *H. pylori*	Esomeprazole 20 mg, bismuth potassium citrate 220 mg, Amo 1,000 mg, Cla 500 mg or Esomeprazole 20 mg, colloidal colloidal bismuth pectin 200 mg Amo, Cla 500 mg, all bid for 14 days	TT: 76.8% (238/310) BQ: 63.69% (100/157)	TT: 83.2% (238/286) BQ: 68.49% (100/146)	TT: 27.1% (84/310) BQ: 37.6% (59/157) 37.6% (59/157)	TT: 100% (310/210) BQ: 100% (157/157)
2019	Qi Chen	China	RCT	*H pylori* positive patients	382	H and C	UBT (at least 6 weeks)	Agar dilution method	Cla 36.4% (104/286), Met 82.2% (235/286), Lev 46.2% (132/286)	Cla 31.3% (30/96), Met 84.4% (81/96), Lev 49.0% (47/96)	Triple therapy or quadruple therapy based on antimicrobial susceptibility to Cla, metronidazole or levofloxacin	Esomeprazole 20 mg and bismuth potassium citrate 600 mg (220 mg elemental bismuth) bid, Amo 1 g and Met 400 mg tid bismuth) bid, Amo 1 g and Met 400 mg tid for 14 days	TT: 91.6% (262/286) BQ: 85.4% (82/96)	TT: 97.7% (250/256) BQ: 97.6% (81/83)	TT: 16.4% (47/286) BQ: 12.5% (12/96)	TT: 94.1% (269/286) BQ: 94.8% (91/96)
2021	Jun-Hyung Cho	Korea	RCT	*H. pylori* positive undergone gastroscopy for epigastric symptoms	282	RUT or DPO-PCR	UBT (at least 4 weeks)	DPO-PCR test	Cla 32.2% (39/141)	NA	Triple therapy or Bismuth-quadruple therapy based on Cla test results	Pantoprazole 40 mg, Amo 1,000 mg, Met 750 mg and Met 750 mg and bismuth subcitrate 600 mg, bid for 14 days	TT: 80.9% (114/141) BQ: 85.8% (121/141)	TT: 89.0% (113/127) BQ: 93.5% (116/124)	TT: 33.8% (44/130) BQ: 29.3% (39/133)	TT: 97.7% (127/130) BQ: 93.2% (124/133)

*W-S staining, Warthin Starry staining; UBT, urea breath test; C, H. pylori culture; H, histology of H. pylori; RUT, Rapid urea test; DPO-PCR, dual priming oligonucleotide polymerase chain reaction; SGT, Susceptibility-guided therapy; BQT, Bismuth Quadruple therapy; Amo, Amoxicillin; Cla, Clarithromycin; Met, Metronidazole; Tet, Tetracycline; Lev, Levofloxacin; Fur, Furazolidon; bid, two times a day; tid, three times a day; qid, four times a day; NA, Not available*.

### Assessment

As demonstrated in Supplementary Figure S1, the 5 RCTs showed a low risk of detection bias, attrition bias, reporting bias, and random sequence generation. Two studies showed an unclear risk in allocation concealment, and three studies showed a low risk. Regarding performance bias, one study showed a high risk, another study showed a low risk, and the remaining three studies showed an unclear risk. As presented in Supplementary Figures S2–S4, there was no significant publication bias in this meta-analysis, as determined by the funnel plot, Begg's test (*p* = 0.24), and Egger's test (*p* = 0.23).

### Comparison of SGT and BQT

#### H. pylori Eradication by ITT and PP

**Figure 2:**
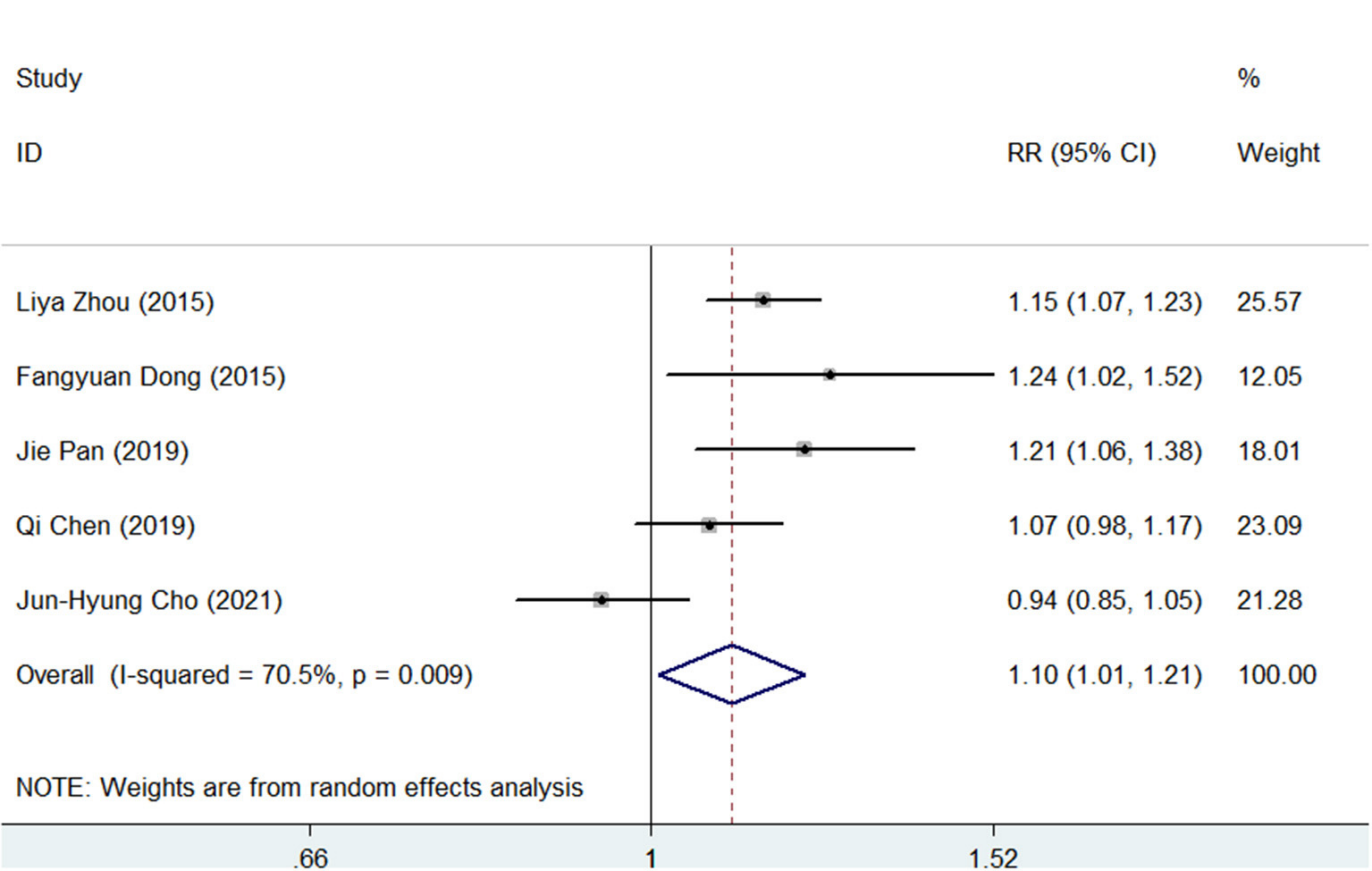
Forest plots for the comparison of susceptibility-guided therapy (SGT) vs. bismuth-containing quadruple therapy (BQT) in *Helicobacter pylori* eradication by an intention-to-treat (ITT) analysis.

As presented in Supplementary Figures S5, S6, the pooled *H. pylori* eradication rate was 86% (95% CI, 80–92) in SGT and 78% (95% CI, 70–85) in BQT by an ITT analysis. As shown in Figure 2, SGT had a superior eradication efficacy compared to BQT, and the difference was statistically significant (pooled RR = 1.10, 95% CI, 1.01–1.21, *p* = 0.03). In addition, a significant heterogeneity (chi-squared test, *p* =0.009, *I*^2^ = 70.5%) was identified between the enrolled studies.

**Figure 3:**
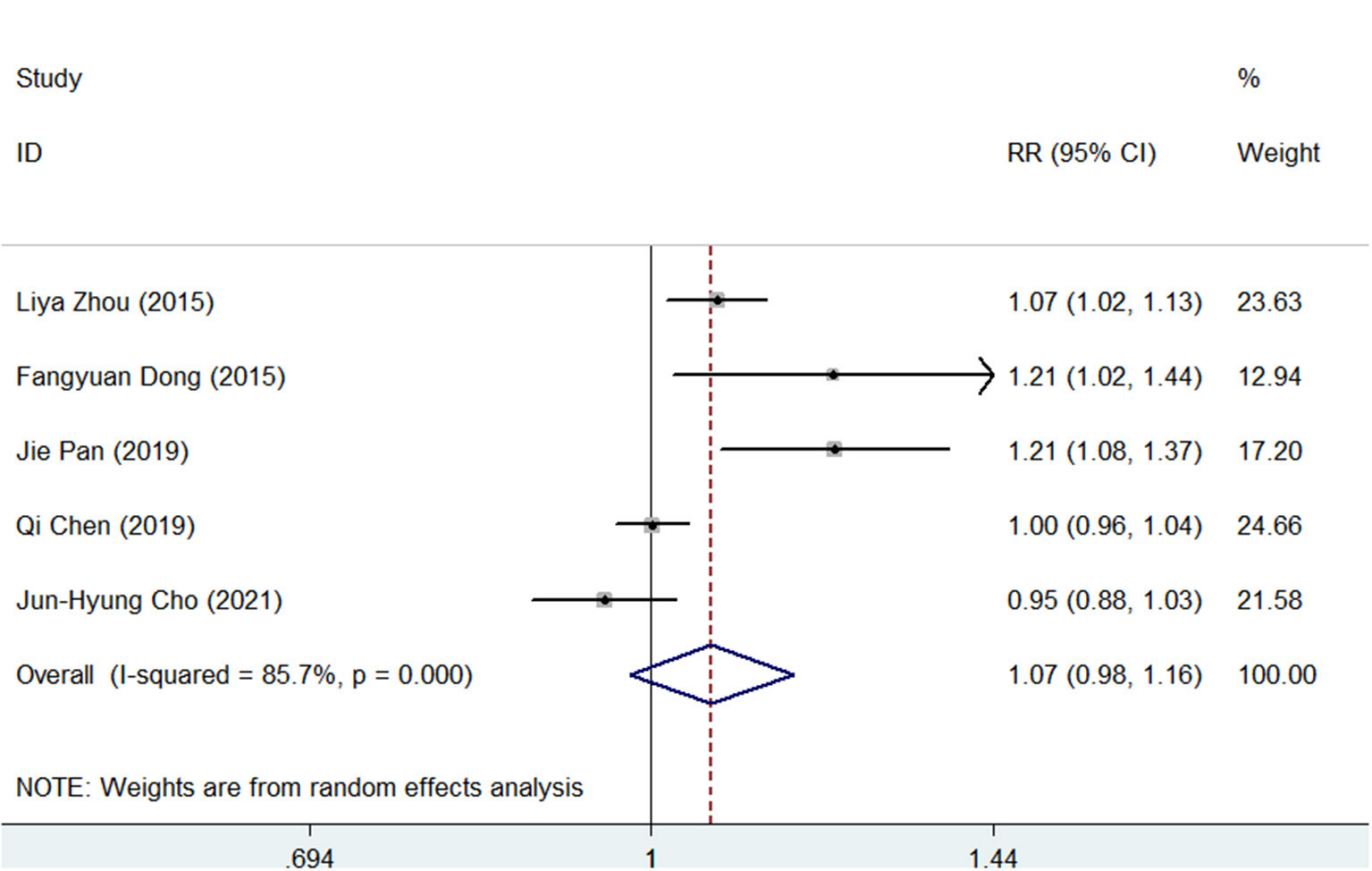
Forest plots for the comparison of SGT vs. BQT in *H. pylori* eradication by a per-protocol (PP) analysis.

For the PP analysis, the pooled *H. pylori* eradication rate was 92% (95% CI, 87–97) in the SGT group and 86% (95% CI, 77–94) in the BQT group (Supplementary Figures S7, S8). As shown in Figure 3, SGT had a superior eradication efficacy compared to BQT although there was no statistical significance (pooled RR = 1.07, 95% CI, 0.98–1.16, *p* = 0.147) and a significant heterogeneity (chi-squared test, *p* = 0, *I*^2^ = 85.7%) was also identified.

Next, we analyzed the eradication rate of SQT and BQT in patients with dual clarithromycin-metronidazole resistance, which was recorded in two studies (17, 18). The eradicate rate was 93% (112/121) in SGT and 80% (93/116) in BQT (SGT vs. BQT; RR = 1.15, 95%CI: 1.02–1.29).

#### Subgroup Analysis

**Figure 4:**
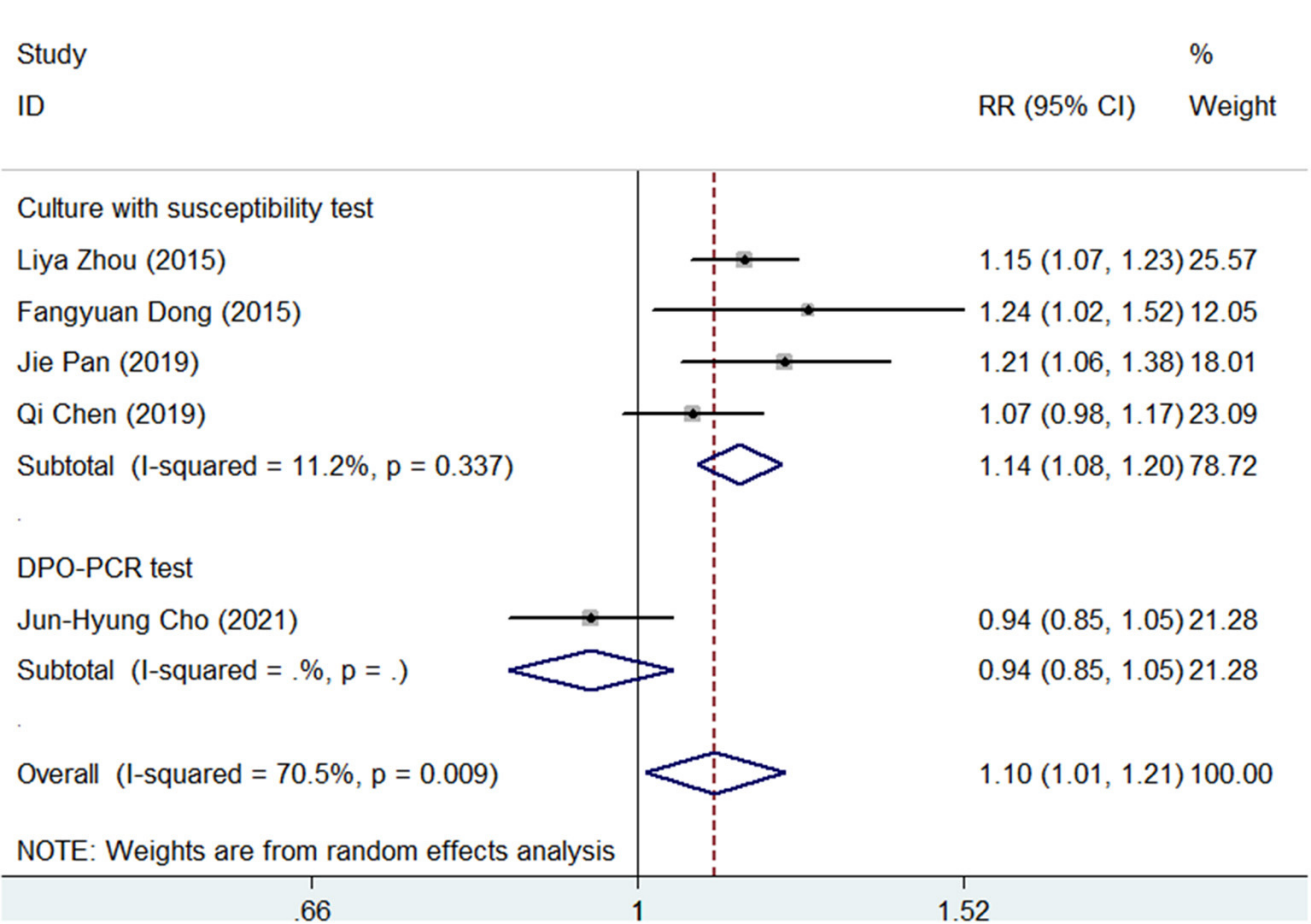
Forest plots for the comparison of SGT vs. BQT using the different types of antimicrobial susceptibility tests in *H. pylori* eradication.

A subgroup analysis was conducted according to the different types of antimicrobial susceptibility tests used in the 5 studies. As shown in Figure 4, SGT had a significantly superior efficacy than BQT (pooled RR = 1.14, 95% CI, 1.08–1.20, *p* < 0.05) in a subgroup of cultures with susceptibility tests. However, in a subgroup of DPO-PCR tests, SGT tended to have a similar efficacy as BQT (RR = 0.94, 95% CI, 0.85–1.05).

Next, we performed a subgroup analysis according to different treatment durations (10 or 14 days) and antibiotic combinations (amoxicillin + clarithromycin or amoxicillin + metronidazole) of BQT in the 5 studies. As shown in Supplementary Figures S9, S10, SGT had a significantly superior efficacy than BQT (pooled RR = 1.16, 95% CI, 1.10–1.23, *p* < 0.05) in the amoxicillin + clarithromycin subgroup, while in the amoxicillin + clarithromycin subgroup, a significant difference was not found (pooled RR = 1.01, 95% CI, 0.89–1.15, *p* > 0.05). For the treatment duration subgroup, SGT showed a superior efficacy than BQT in both 10- (pooled RR = 1.15, 95% CI, 1.07–1.23) and 14-day (pooled RR = 1.09, 95% CI, 0.97–1.23) subgroups.

#### Side Effects

**Figure 5:**
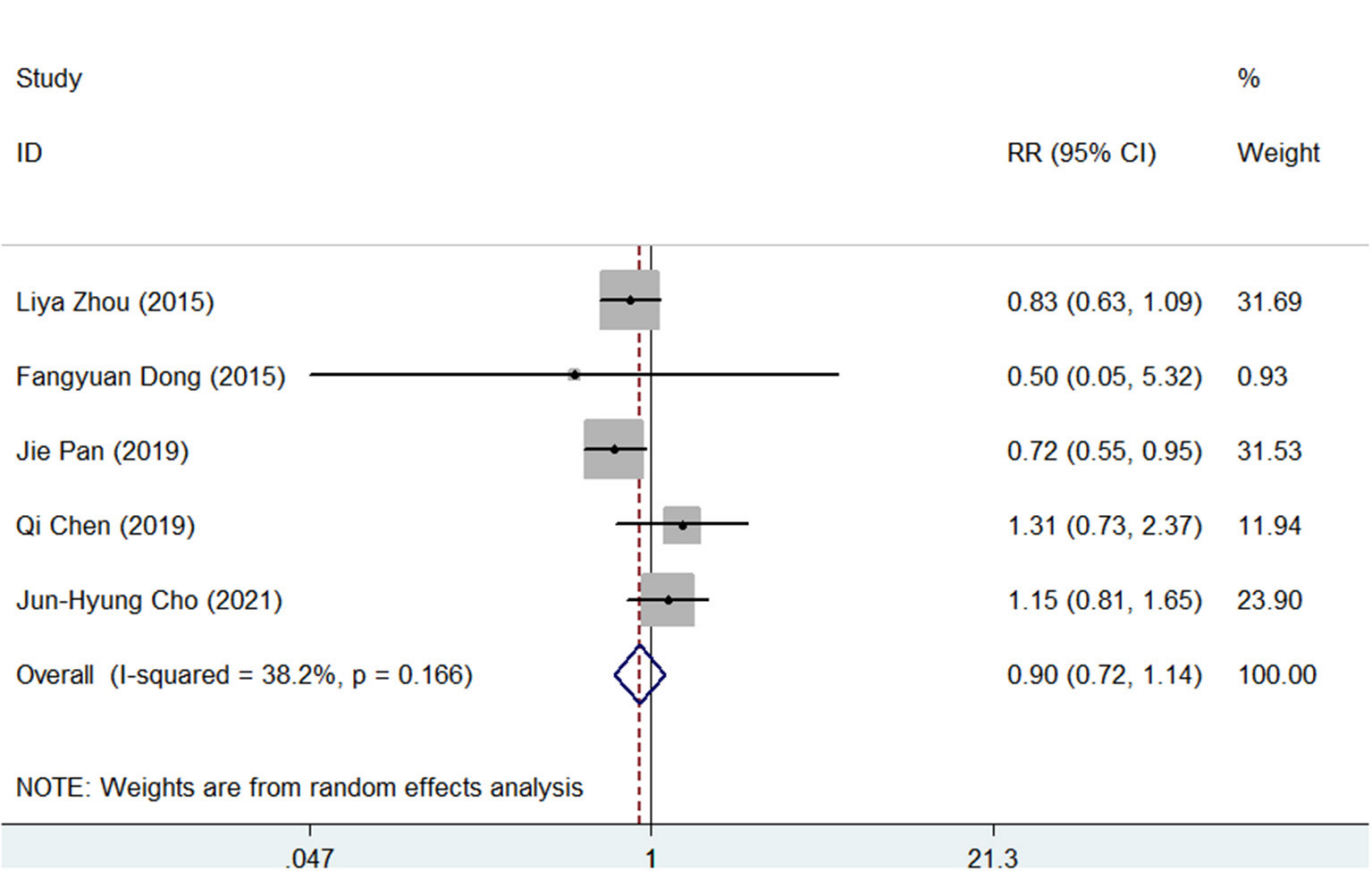
Forest plots for the comparison of SGT vs. BQT in side effects.

The side effects were recorded in all studies. As shown in Supplementary Figures S11, S12, the pooled side effect rate was 20% (95% CI, 10–30) in SGT and 22% (95% CI, 11–33) in BQT. There were no significant differences between the two groups regarding the side effects (RR = 0.90, 95% CI, 0.72–1.14, *p* = 0.383), as presented in Figure 5.

#### Compliance

**Figure 6:**
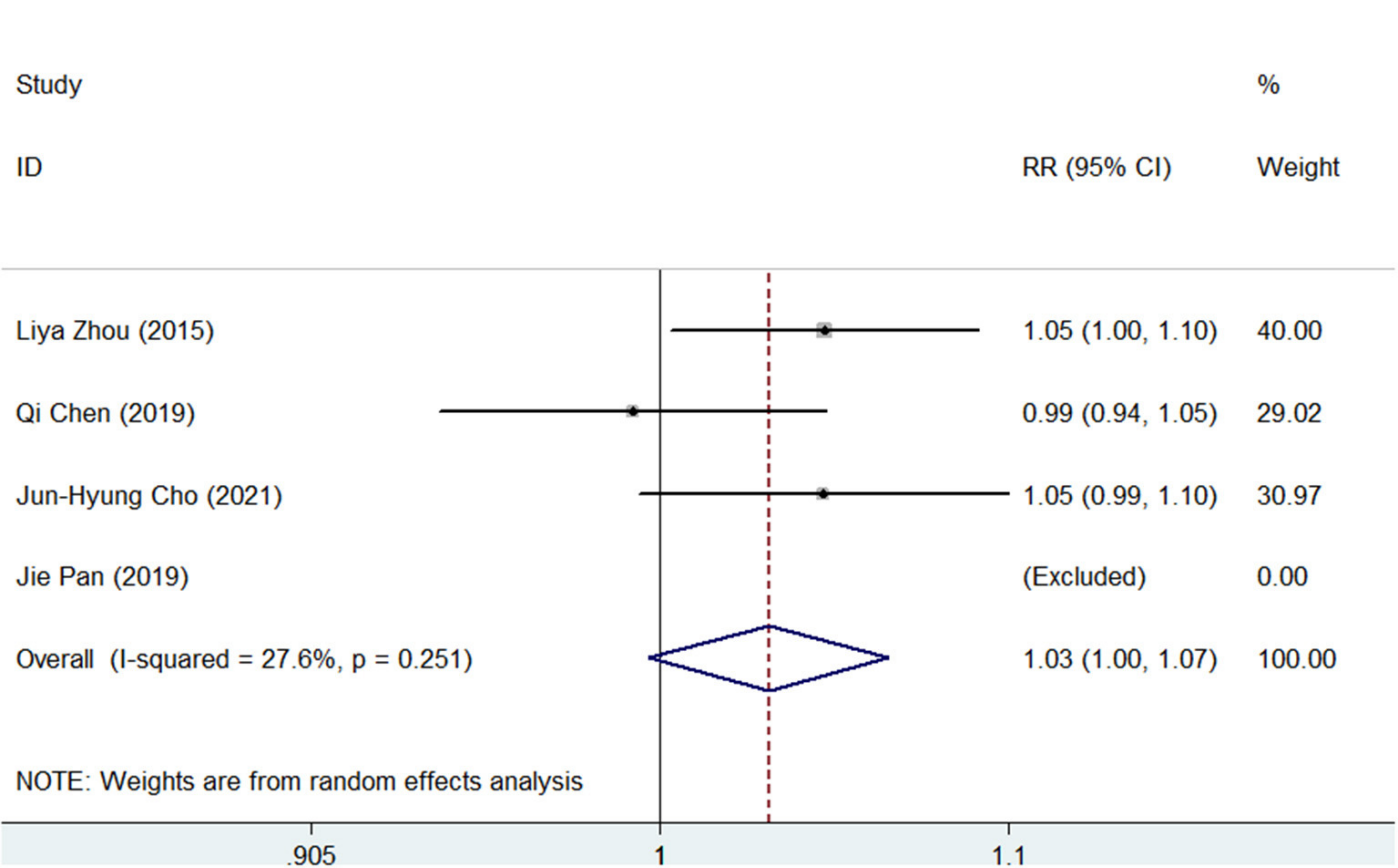
Forest plots for the comparison of SGT vs. BQT in compliance.

Compliance was recorded in 4 of the 5 studies and defined as patients who took more than 80% of the treatment drugs. As shown in Supplementary Figures S13, S14, the pooled compliance rate was 95.0% (95% CI, 93–98) in SGT and 92% (95% CI, 89–95) in BQT. As shown in Figure 6, although SGT tended to have a higher rate of compliance vs. BQT, there was no significant difference (RR = 1.03, 95% CI, 1.00–1.07, *p* = 0.07).

## Discussion

The susceptibility-guided therapy of *H. pylori* can be based on the results of culture with susceptibility tests or a molecular determination of genotype resistance, which is recommended after the failure of the second-line treatment in the Maastricht V/Florence Consensus Report (4). *H. pylori* resistance to antibiotics has become a serious global problem in recent years, mainly because of increased antibiotic use in the community (7, 24). Rational first-line regimens with a high efficacy should be applied to eradicate *H. pylori* to avoid secondary antibiotic resistance and failures of second- or third-line treatment, especially in areas with high antibiotic resistance. Theoretically, a susceptibility test is the best choice for guiding a tailored therapy for *H. pylori* infection. If not available, empiric BQT is recommended, and sensitive antibiotic combinations should be used in the regimens based on the region or population-specific antibiotic susceptibility data (13, 25). A recent Taipei global consensus also emphasized the importance of susceptibility testing for guiding decision-making for *H. pylori* regimens (5).

A previous meta-analysis (26–28) was conducted to explore a tailored therapy vs. an empirically chosen treatment for *H. pylori* eradication, which showed higher efficacy of the tailored therapy. The empiric treatments included standard or quadruple therapies, and standard therapy was not recommended for eradicating *H. pylori* because of its high resistance rate. Moreover, heterogeneity might exist because first-line or rescue treatments were included. DPO-PCR is a novel method applied in clinical practice. As such, this updated systematic review and meta-analysis included 5 RCTs to evaluate the efficacy of SGT vs. BQT for 10 or 14 days (recommended by the current consensus reports) as the first-line treatment for *H. pylori*. In total, SGT showed higher efficacy than BQT according to the ITT and PP analysis because the resistance rate of *H. pylori* was high in the included studies. We further conducted a subgroup analysis according to the different types of antimicrobial susceptibility tests of SGT, treatment durations, and BQT antibiotic combinations. Interestingly, SGT had a significantly superior efficacy than BQT in a subgroup of cultures with susceptibility tests. Cultures with susceptibility tests included the *E*-test, agar dilution test, and disk diffusion method. The agar dilution method is considered the gold standard for detecting the antibiotic resistance of *H. pylori* according to the Clinical and Laboratory Standards Institute (29). The *E*-test (different concentrations of antibiotics in a single strip were used) is simpler, has lower cost, and is less time-consuming than the agar dilution method (30), which is an acceptable alternative for antimicrobial susceptibility testing for *H. pylori*. Several studies (31–33) evaluated the accuracy of the *E*-test for *H. pylori* antibiotic resistance using the agar dilution method as the gold standard, and the results showed an acceptable agreement between the two methods. The *E*-test or agar dilution test can provide accurate antibiotic resistance information for *H. pylori*, guiding a tailored therapy for eradicating *H. pylori*. As such, a higher eradication rate of SGT was observed in comparison with BQT in a subgroup analysis of cultures with susceptibility tests.

Considering the time-consuming, cost-effectiveness, and false-negative results of culture and clarithromycin being the most common antibiotics in the first-line regimens for *H. pylori*, DPO-PCR is applied in clinical practice because of its straightforward and rapid test for *H. pylori* infection and clarithromycin resistance (34). A2142G and A2143G mutations were most frequent and account for ~80–90% of clarithromycin resistance (35). If mutations exist, the other antibiotics will be used in the *H. pylori* regimens. Otherwise, clarithromycin will be used in the regimens for eradicating *H. pylori*. Our results showed no differences in the eradication rate between SGT and BQT in a subgroup analysis of DPO-PCR. Several factors might explain this result: (1) Other mutations (36–39) of clarithromycin resistance also exist, including A2142C, A2115G, and G2141A. (2) The A2142C point mutation was reported to show a higher efficacy than A2142G and A2143G for inducing clarithromycin resistance (40). (3) DPO-PCR only provides information on clarithromycin resistance, which could not guide the choice of other antibiotics used in the *H. pylori* regimens.

Currently, the prevalence of *H. pylori* primary resistance to clarithromycin is high (7, 24), especially in China. Clarithromycin-resistant *H. pylori* is classified by the WHO as a high-priority bacterium (8). Moreover, clarithromycin resistance cannot be overcome by increasing the dose, frequency, or duration of the antibiotic. As such, SGT has significantly superior efficacy than BQT in the amoxicillin + clarithromycin combination subgroup.

Side effects and compliance were also analyzed in our study, and the results showed that the side effects of both groups were below 25%, and no serious side effects occurred. These data indicate the safety of SGT and BQT in the clinical practice of *H. pylori* eradication. Compliance is an important factor influencing the eradication rate of *H. pylori*. Several studies (41–43) were conducted in China to evaluate useful methods to improve compliance in the management of *H. pylori* infection. Interestingly, short-message-based reeducation or WeChat platform (a social media platform) reminders two times daily were the efficient ways to improve the eradication rate of *H. pylori* infection and elevate the compliance of *H. pylori* regimens. Our results also showed good compliance of SGT and BQT in the management of *H. pylori* infection.

There are limitations regarding this review. First, the included studies were conducted in China and Korea and also in areas with a high resistance rate to antibiotics, which may have limited the applicability of these findings outside Asian countries. The comparison between SGT and BQT in areas with moderate or low resistance rates to antibiotics remains unclear. Second, the results were not applied for patients who were allergic to penicillin. Third, the sample size of the included studies was limited, especially in a subgroup analysis. Fourth, heterogeneity existed when data from different studies were combined, which were probably attributed to the regimens, the method to detect antibiotic resistance, and the populations included in the enrolled studies. Fifth, our study aimed to compare the efficacy of SGT and BQT as the first-line treatment of *H. pylori*, which could not be applied in the second- or third-line treatment of *H. pylori*. Sixth, an unclear risk in allocation concealment and performance bias existed in a subset of the included studies, and a selection bias might have existed in this study. Seventh, the studies that did not mention the keywords in titles/abstracts or were published in other languages might have been missed in this review.

In conclusion, compared with BQT, SGT showed a higher efficacy and similar safety as the first-line treatment of *H. pylori* infection in areas with high antibiotic resistance. The decision-making of first-line regimens for *H. pylori* infection should depend on the availability and cost-effectiveness of susceptibility tests and bismuth in local areas, which is consistent with the recommendations of the Taipei global consensus (5).

## Author Contributions

YH was guarantor of the article. YO, WZ, and CH performed the literature search with a systematic review and meta-analysis and extracted the data. YO and YH wrote the manuscript. YZ, NL, and YH designed a systematic review and edited this manuscript. All authors approved the final version of this manuscript.

## Funding

This study was supported by the National Natural Science Foundation of China (No. 82000531), the Project for Academic and Technical Leaders of Major Disciplines in Jiangxi Province (No. 20194BCJ22016), the Key Research and Development Program of Jiangxi Province (No. 20212BBG73018), the Youth Project of the Jiangxi Natural Science Foundation (No. 20202BABL216006), the Key Fund of the Jiangxi Education Department (No. GJJ190007), the Scientific Research of Health Commission of Jiangxi Province (No. 20213019), Scientific Research of Traditional Chinese Medicine of Jiangxi Province (No. 2020A0047), and the Young Teachers' Scientific Research and Cultivation Fund of the Medical Department of Nanchang University (PY201919).

## Conflict of Interest

The authors declare that the research was conducted in the absence of any commercial or financial relationships that could be construed as a potential conflict of interest.

## Publisher's Note

All claims expressed in this article are solely those of the authors and do not necessarily represent those of their affiliated organizations, or those of the publisher, the editors and the reviewers. Any product that may be evaluated in this article, or claim that may be made by its manufacturer, is not guaranteed or endorsed by the publisher.

## Supplementary Material

Supplementary Figure S1 Quality assessment of randomized controlled trials (RCT) studies.

Supplementary Figure S2 Publication bias evaluated by a funnel plot.

Supplementary Figure S3 Publication bias evaluated by Begg's test.

Supplementary Figure S4 Publication bias evaluated by Egger's test.

Supplementary Figure S5 Forest plots for the pooled eradication rate of susceptibility-guided therapy (SGT) by an intention-to-treat (ITT) analysis.

Supplementary Figure S6 Forest plots for the pooled eradication rate of bismuth-containing quadruple therapy (BQT) by an ITT analysis.

Supplementary Figure S7 Forest plots for the pooled eradication rate of SGT by per-protocol (PP) analysis.

Supplementary Figure S8 Forest plots for the pooled eradication rate of BQT by PP analysis.

Supplementary Figure S9 Forest plots for the comparison of SGT vs. BQT by different treatment duration in *Helicobacter pylori* eradication.

Supplementary Figure S10 Forest plots for the comparison of SGT vs. BQT by different antibiotic combinations in *H. pylori* eradication.

Supplementary Figure S11 Forest plots for the pooled side effect rate of SGT.

Supplementary Figure S12 Forest plots for the pooled BQT side effect rate.

Supplementary Figure S13 Forest plots for the pooled compliance of SGT.

Supplementary Figure S14 Forest plots for the pooled compliance of BQT.

Supplementary Table S1 Full search strategy in PubMed, Embase, and Cochrane Central.

Supplementary Table S2 The PRISMA 2009 checklist.
