# Supplementary figures and images for: Susceptibility-Guided Therapy vs. Bismuth-Containing Quadruple Therapy as the First-Line Treatment for Helicobacter pylori Infection: A Systematic Review and Meta-Analysis
Source: Front Med (Lausanne). 2022 Mar 24;9:844915. doi: 10.3389/fmed.2022.844915 (PMC8987208; doi:10.3389/fmed.2022.844915)

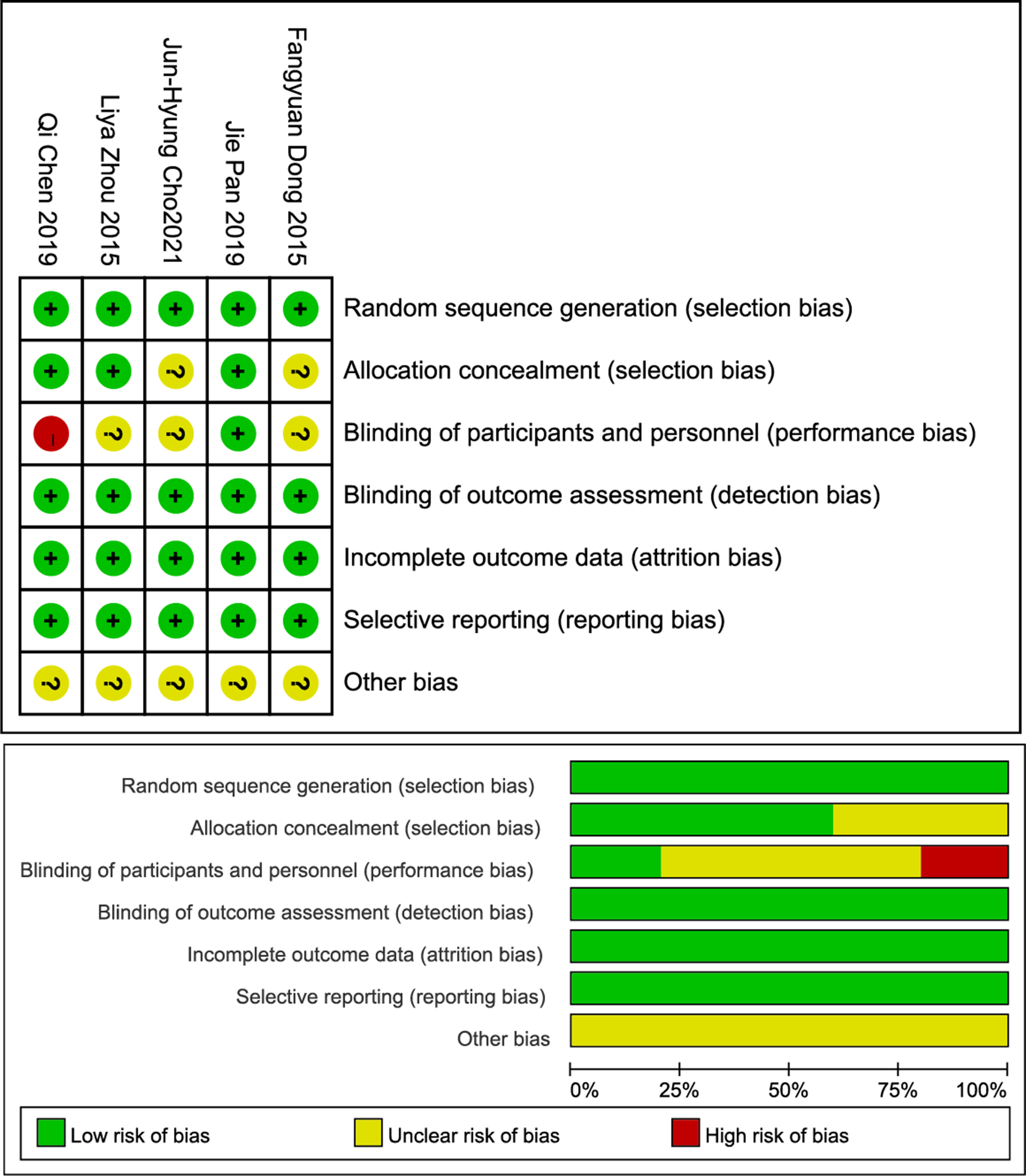

Supplement: Supplementary Figure S1 — Quality assessment of randomized controlled trials (RCT) studies. [file Image_1.TIF]

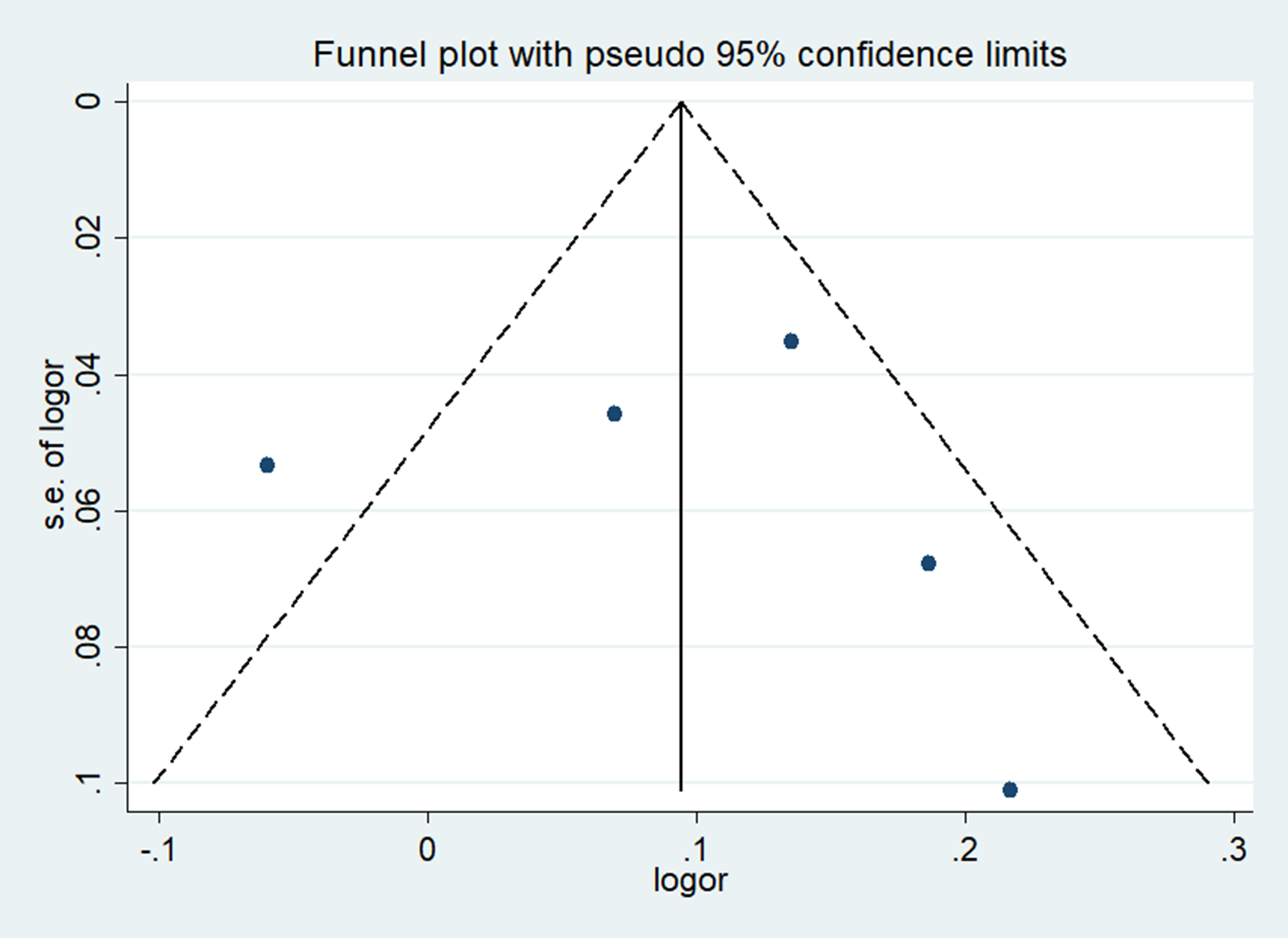

Supplement: Supplementary Figure S2 — Publication bias evaluated by a funnel plot. [file Image_2.TIF]

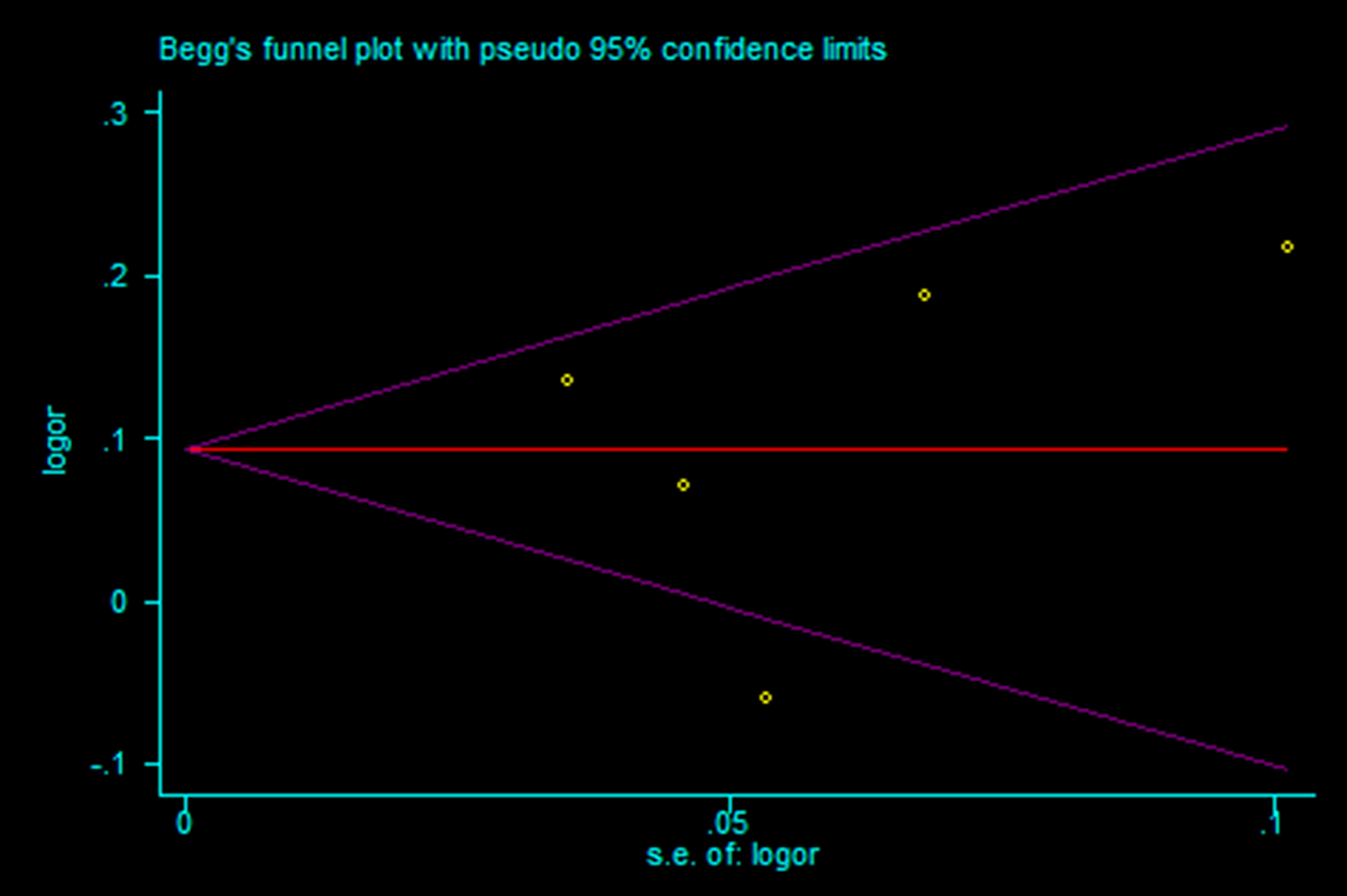

Supplement: Supplementary Figure S3 — Publication bias evaluated by Begg's test. [file Image_3.TIF]

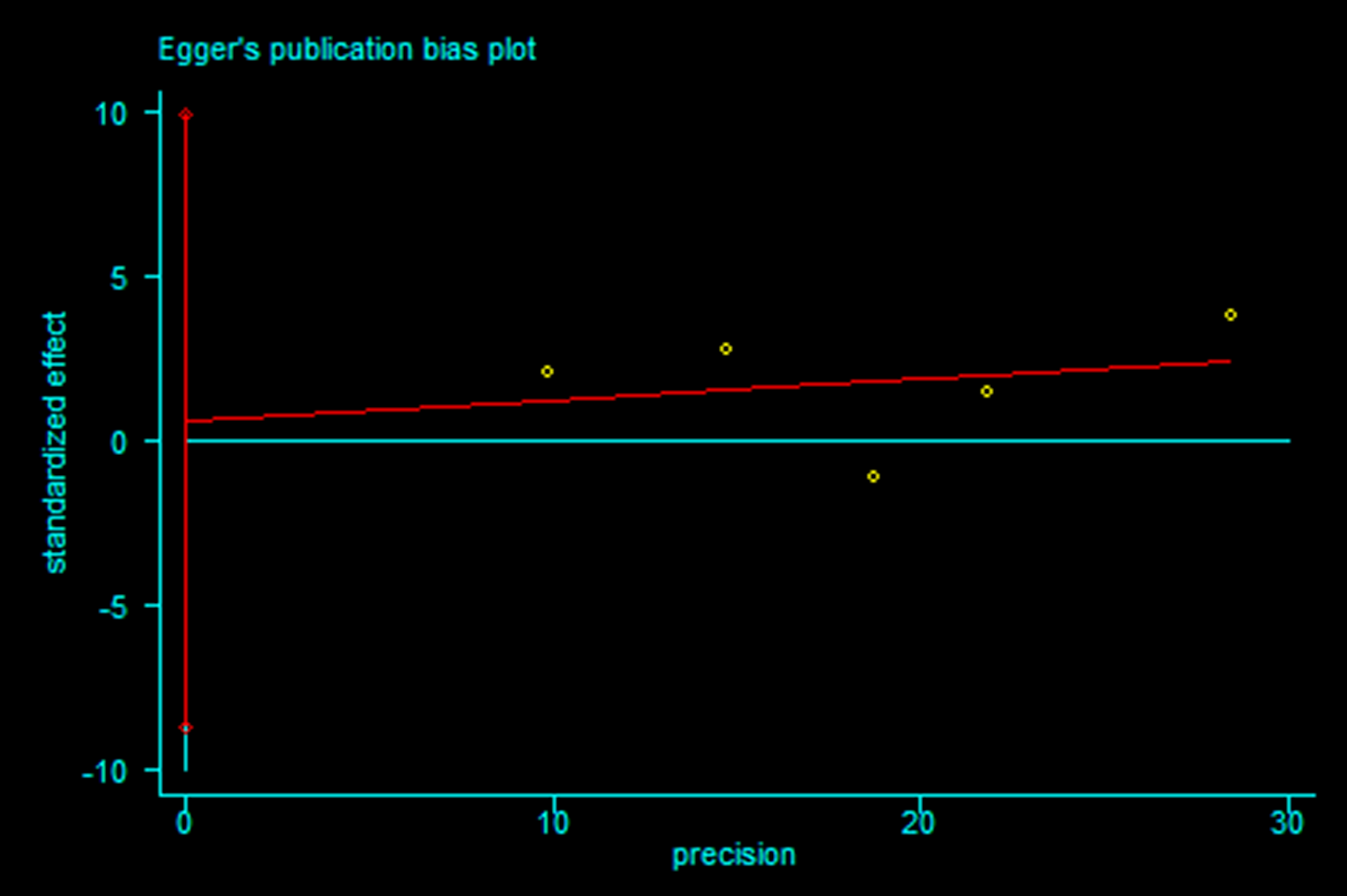

Supplement: Supplementary Figure S4 — Publication bias evaluated by Egger's test. [file Image_4.TIF]

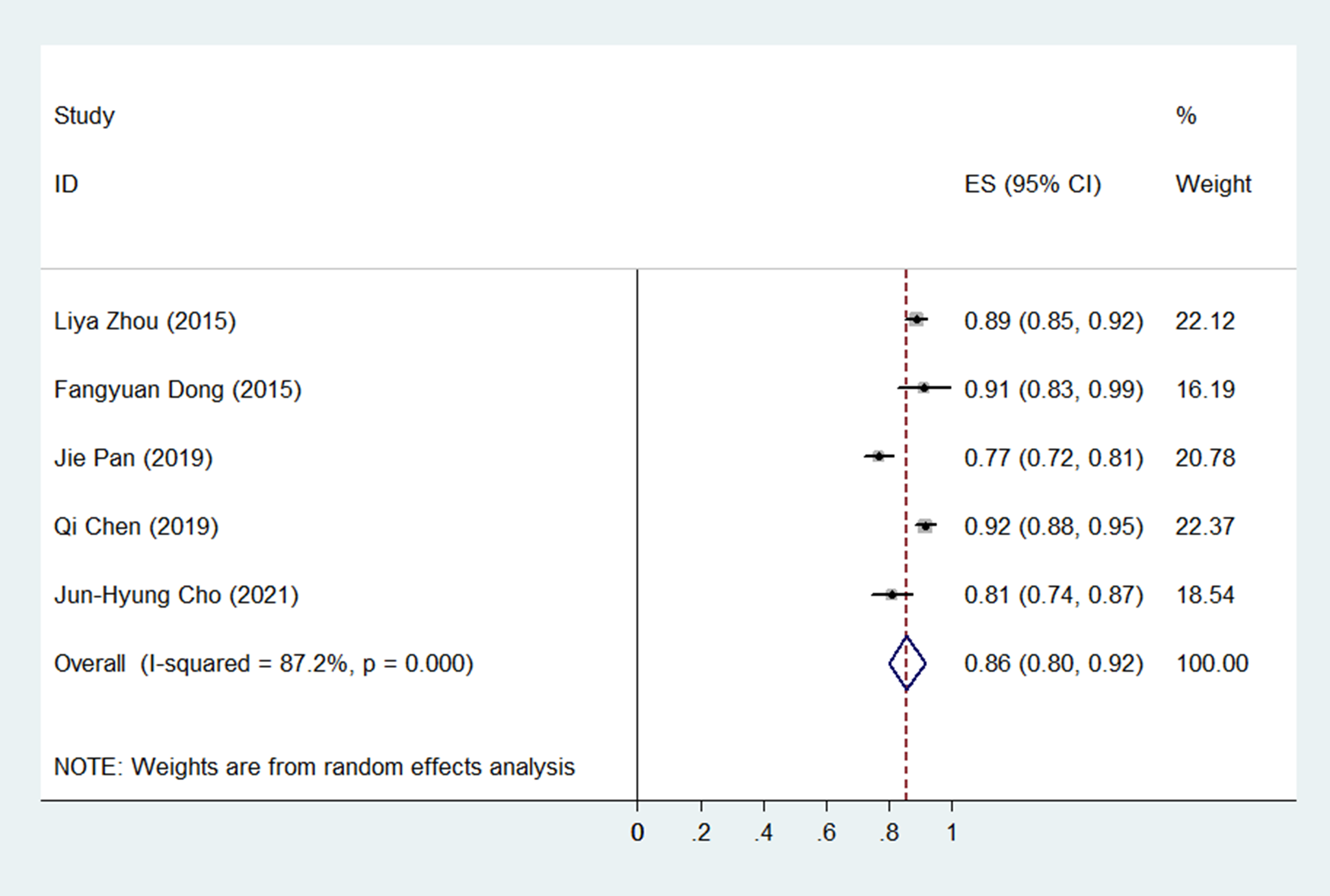

Supplement: Supplementary Figure S5 — Forest plots for the pooled eradication rate of susceptibility-guided therapy (SGT) by an intention-to-treat (ITT) analysis. [file Image_5.TIF]

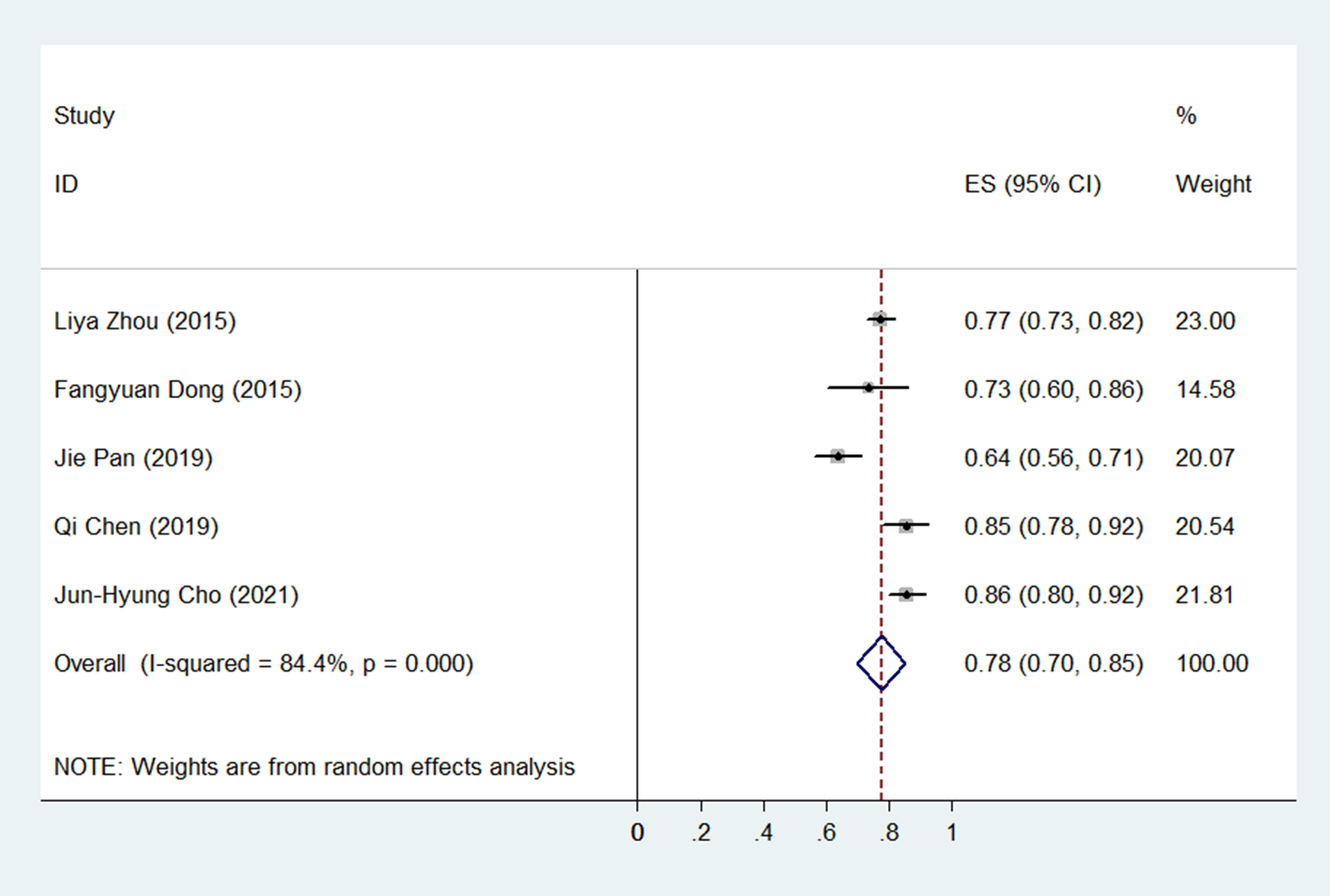

Supplement: Supplementary Figure S6 — Forest plots for the pooled eradication rate of bismuth-containing quadruple therapy (BQT) by an ITT analysis. [file Image_6.TIF]

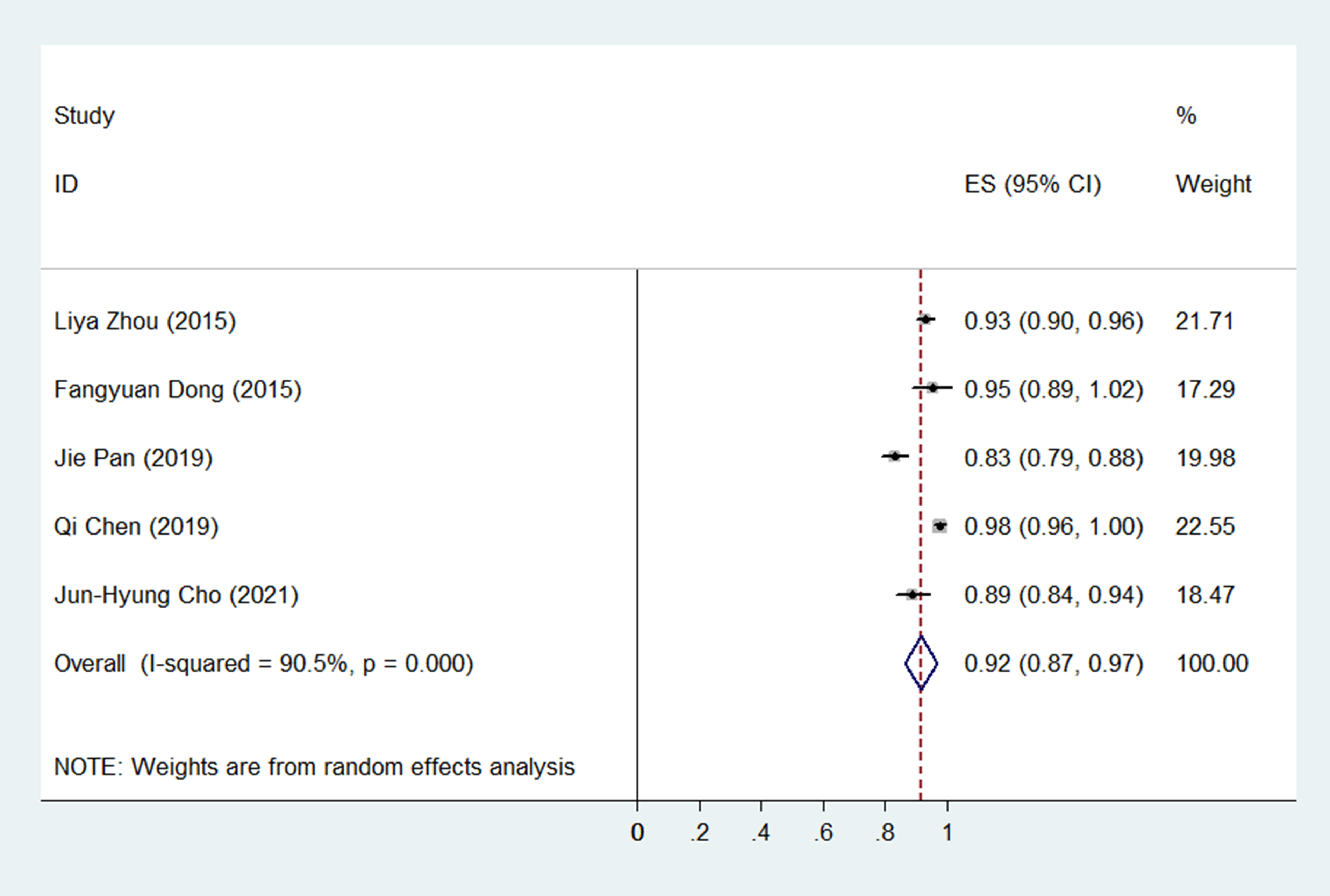

Supplement: Supplementary Figure S7 — Forest plots for the pooled eradication rate of SGT by per-protocol (PP) analysis. [file Image_7.TIF]

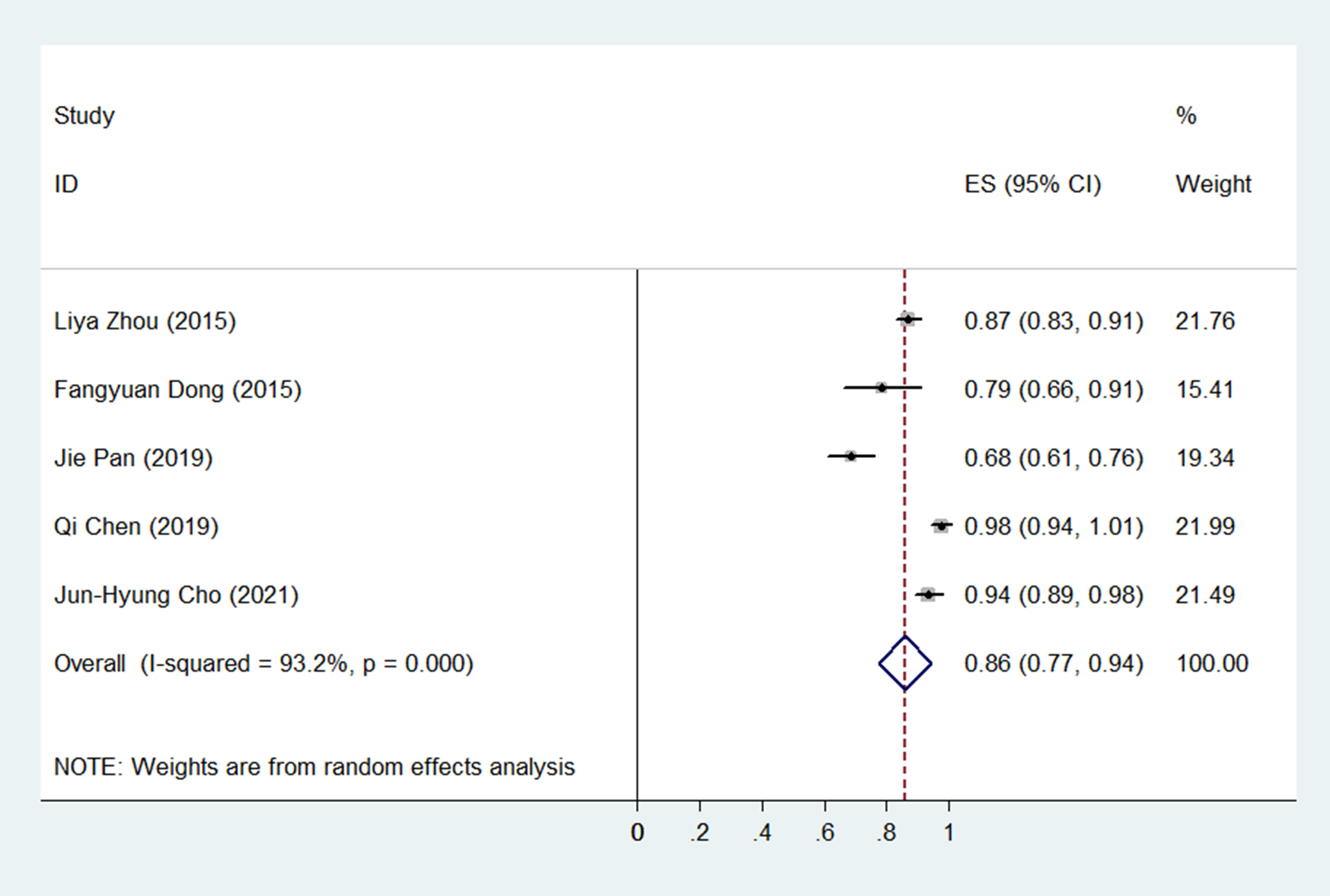

Supplement: Supplementary Figure S8 — Forest plots for the pooled eradication rate of BQT by PP analysis. [file Image_8.TIF]

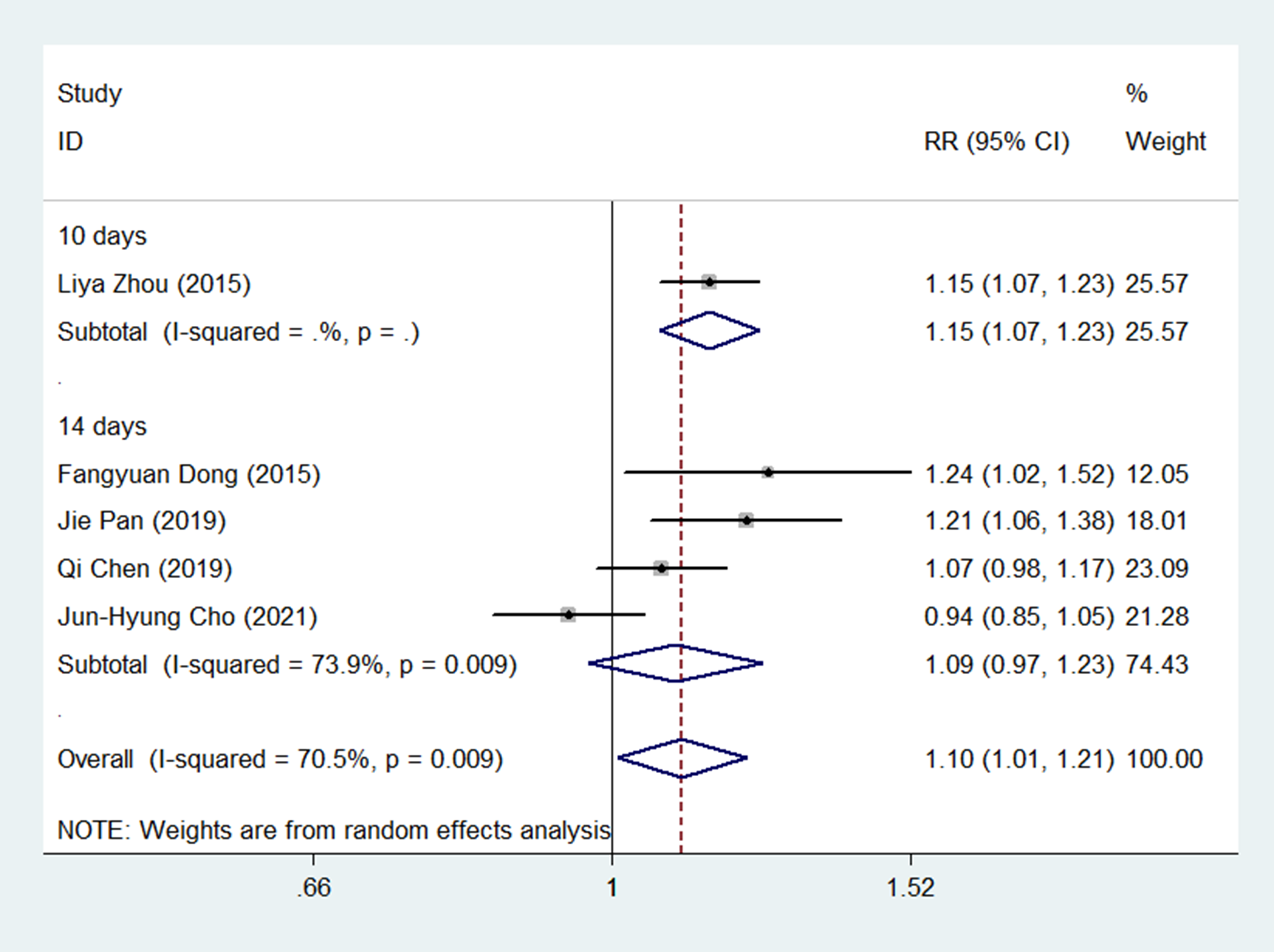

Supplement: Supplementary Figure S9 — Forest plots for the comparison of SGT vs. BQT by different treatment duration in Helicobacter pylori eradication. [file Image_9.TIF]

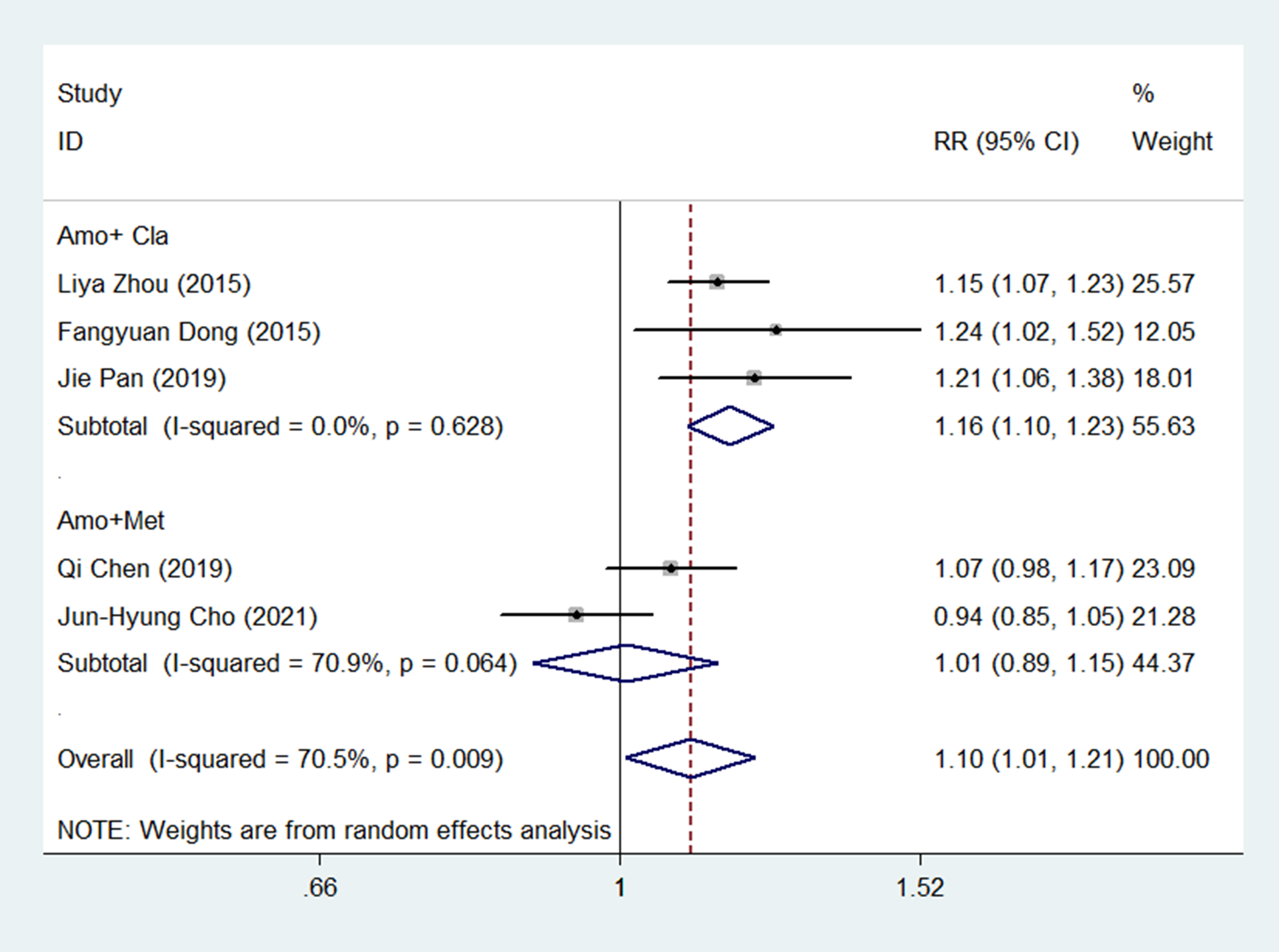

Supplement: Supplementary Figure S10 — Forest plots for the comparison of SGT vs. BQT by different antibiotic combinations in H. pylori eradication. [file Image_10.TIF]

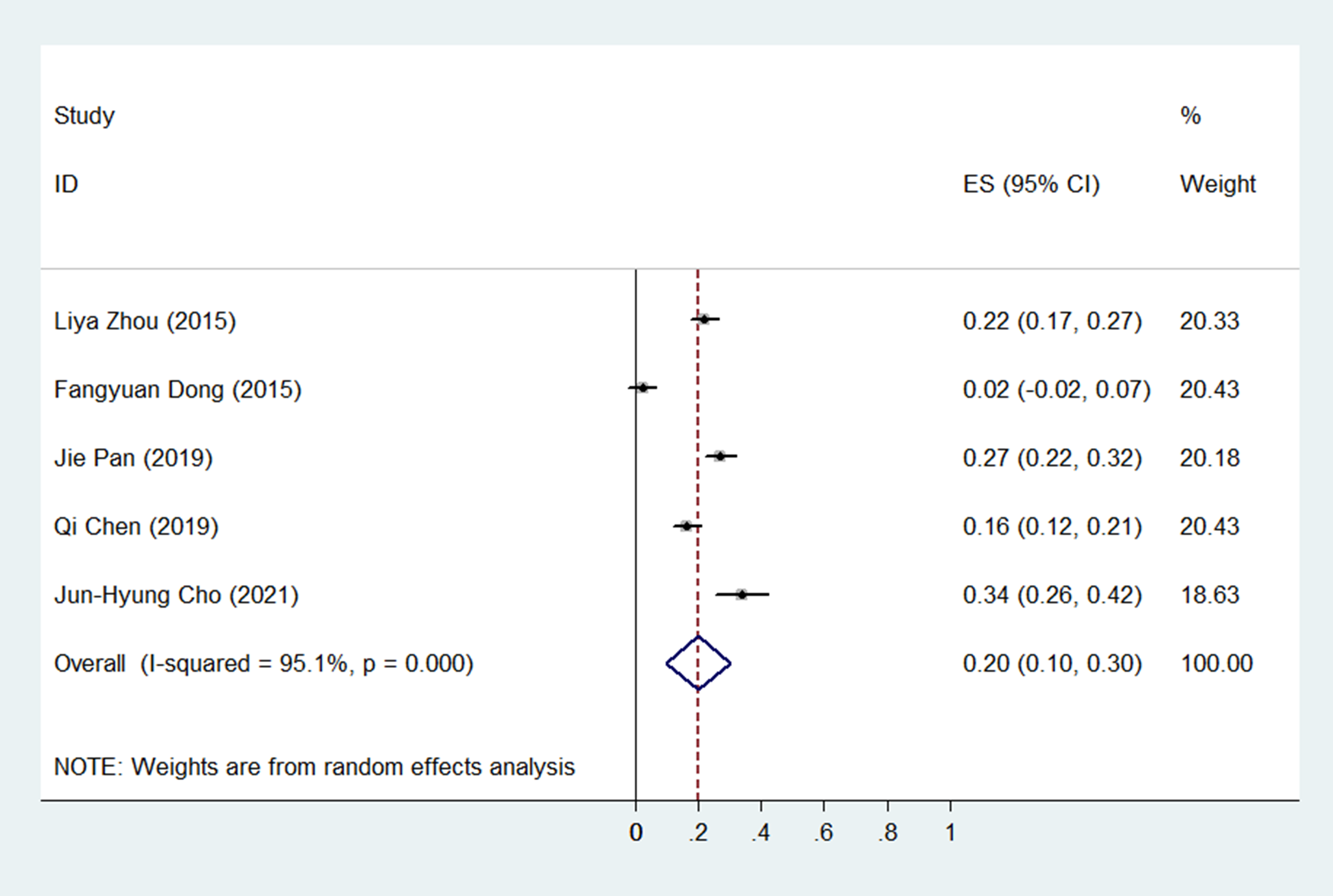

Supplement: Supplementary Figure S11 — Forest plots for the pooled side effect rate of SGT. [file Image_11.TIF]

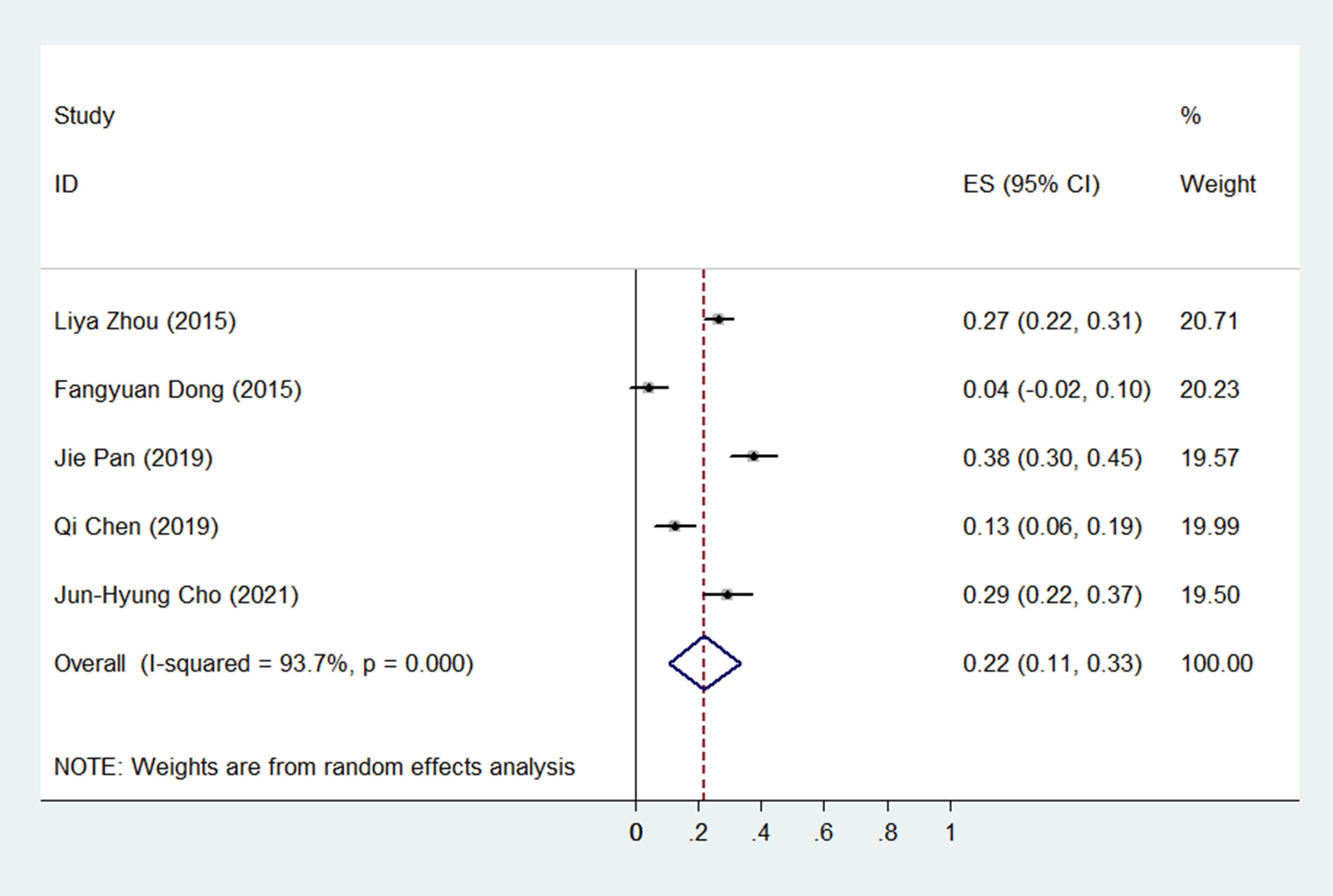

Supplement: Supplementary Figure S12 — Forest plots for the pooled BQT side effect rate. [file Image_12.TIF]

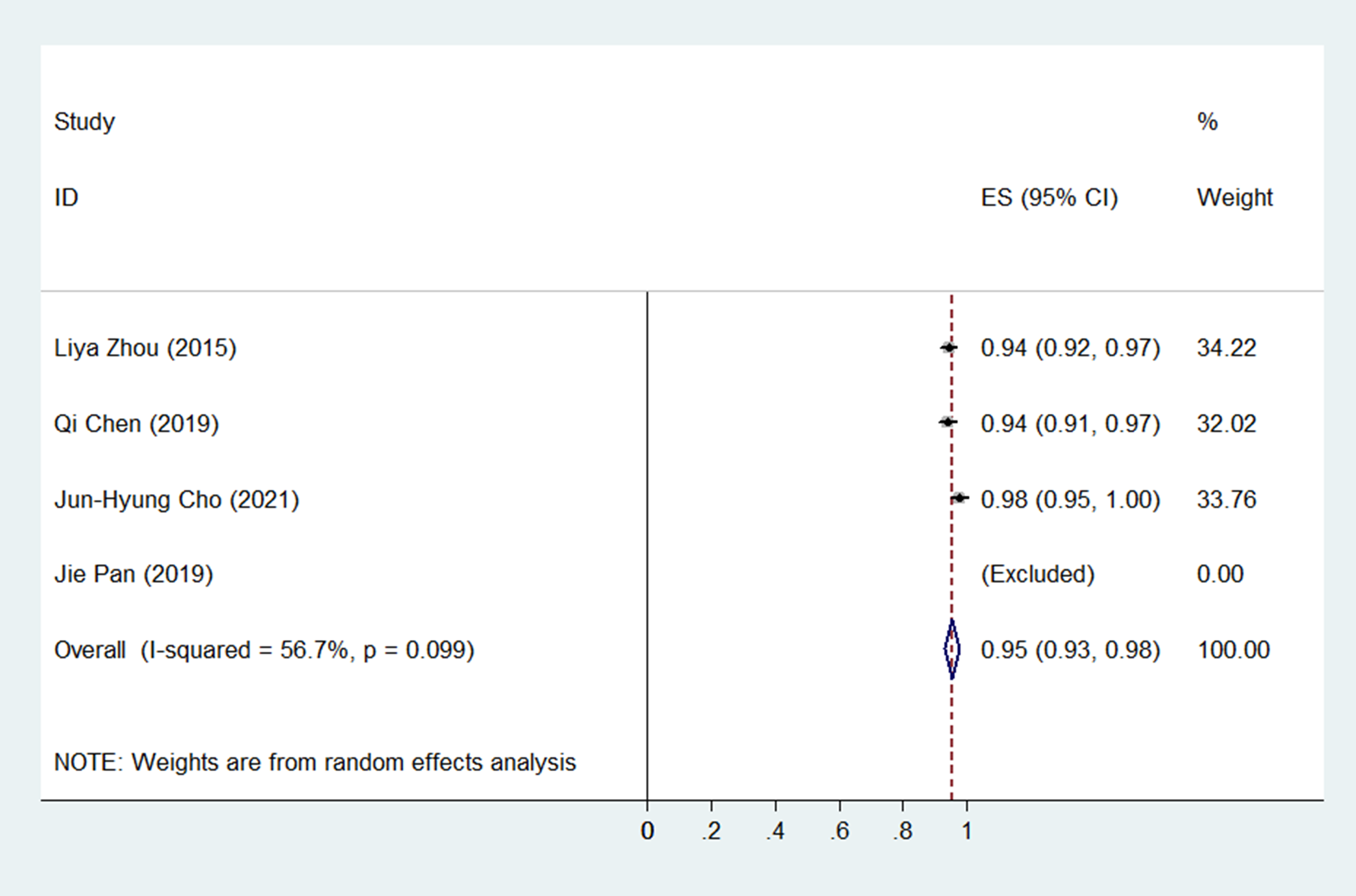

Supplement: Supplementary Figure S13 — Forest plots for the pooled compliance of SGT. [file Image_13.TIF]

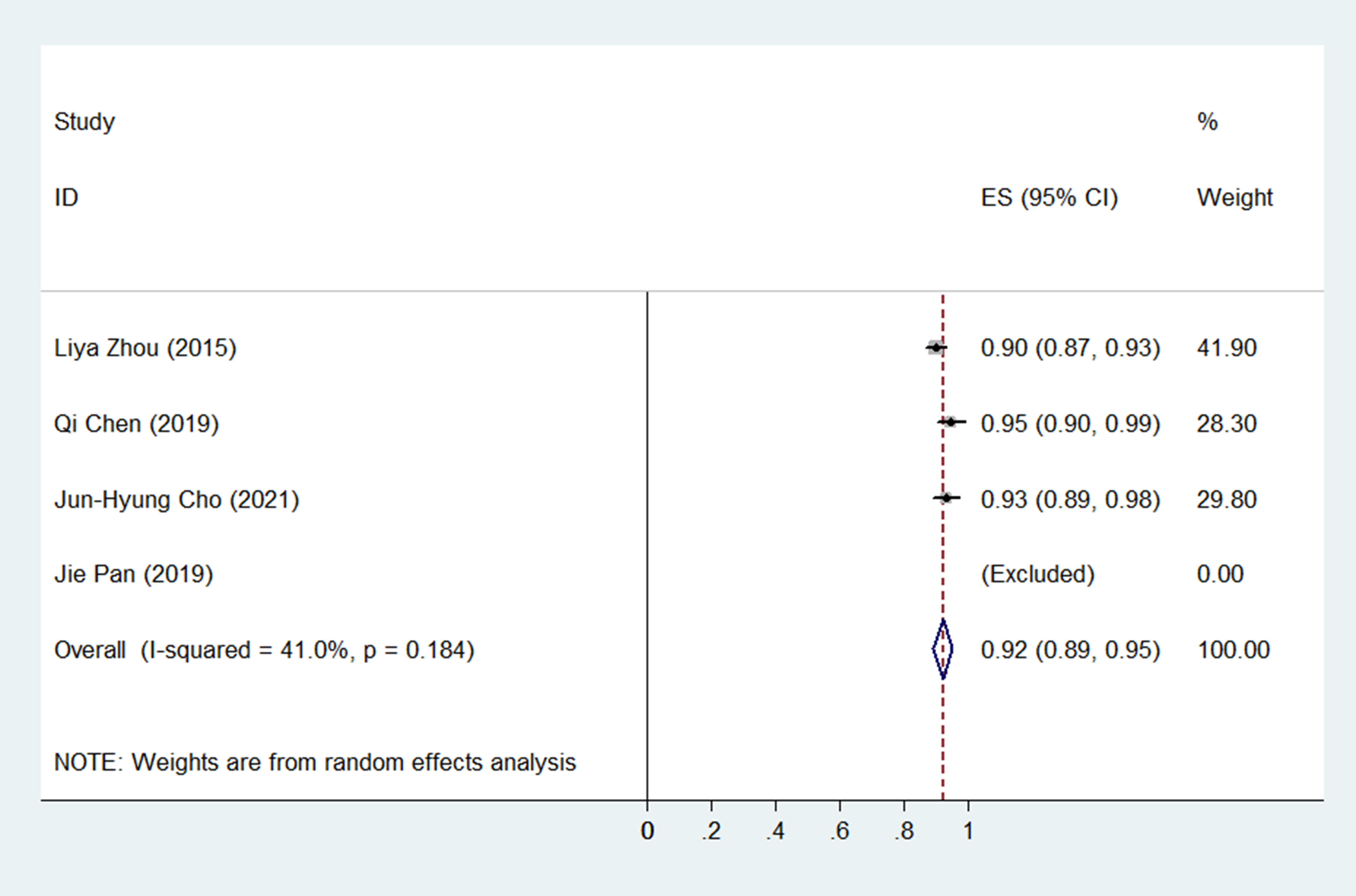

Supplement: Supplementary Figure S14 — Forest plots for the pooled compliance of BQT. [file Image_14.TIF]
