# Supplementary material for: Susceptibility-Guided Therapy vs. Bismuth-Containing Quadruple Therapy as the First-Line Treatment for Helicobacter pylori Infection: A Systematic Review and Meta-Analysis
Source: Front Med (Lausanne). 2022 Mar 24;9:844915. doi: 10.3389/fmed.2022.844915 (PMC8987208; doi:10.3389/fmed.2022.844915)
Supplement: Supplementary Table S1 — Full search strategy in PubMed, Embase, and Cochrane Central. [file Table_1.docx]

Table S1: Full search strategy in Pubmed, Embase and Cochrane Central Register of Controlled Trials

| Search | Query | Items found |
| --- | --- | --- |
| #4 | Search (Tailored eradication OR tailored therapy OR susceptibility-guided therapy OR personalized therapy OR antibiotic susceptibility testing) AND (Helicobacter pylori OR H. pylori) AND bismuth quadruple | 497 |
| #3 | Search bismuth quadruple | 805 |
| #2 | Search (Helicobacter pylori OR H. pylori) | 48087 |
| #1 | Search (Tailored eradication OR tailored therapy OR susceptibility-guided therapy OR personalized therapy OR antibiotic susceptibility testing) | 5354259 |

S1.1 Pubmed database search

S1.2 Cochrane Central Register of Controlled Trials search

| ID | Search | Hits |
| --- | --- | --- |
| #1 | (Helicobacter pylori OR H. pylori) | 2028 |
| #2 | (Tailored eradication OR tailored therapy OR susceptibility-guided therapy OR personalized therapy OR antibiotic susceptibility testing) | 9630 |
| #3 | bismuth quadruple | 683 |
| #4 | #1 AND #2 AND #3 | 97 |

S1.3 Embase search

| No. | Query | Results |
| --- | --- | --- |
| #4 | #1 AND #2 AND #3 | 62 |
| #3 | bismuth quadruple | 1561 |
| #2 | 'helicobacter pylori'/exp | 57308 |
| #1 | (Tailored eradication OR tailored therapy OR susceptibility-guided therapy OR personalized therapy OR antibiotic susceptibility testing) | 19861 |
